# Editorial: Development of small molecule inhibitors and antibodies targeting AXL for tumor therapy and infectious disease control

**DOI:** 10.3389/fonc.2022.1121120

**Published:** 2023-01-10

**Authors:** Kyoung-Ho Pyo, S. M. Mazidur Rahman, Diana Boraschi

**Affiliations:** ^1^ Severance Biomedical Science Institute, Yonsei University College of Medicine, Seoul, South Korea; ^2^ Yonsei New Il Han Institute for Integrative Lung Cancer Research, Yonsei University College of Medicine, Seoul, South Korea; ^3^ International Centre for Diarrhoeal Disease Research (iccdr,b), Dhaka, Bangladesh; ^4^ Shenzhen Institute of Advanced Technology (SIAT), Chinese Academy of Sciences (CAS), Shenzhen, China; ^5^ Institute of Biochemistry and Cell Biology, National Research Council (CNR), Napoli, Italy; ^6^ Stazione Zoologica Anton Dohrn (SZN), Napoli, Italy; ^7^ China-Italy Joint Laboratory of Pharmacobiotechnology for Medical Immunomodulation, Shenzhen, China

**Keywords:** AXL, small molecule inhibitors, antibodies, tumor therapy, therapy of infections

For a long time, anti-cancer therapeutic strategies have mainly addressed the tumor, in the attempt to subvert the altered signaling pathways leading to oncogenesis (1, 2). It is however evident that the tumor microenvironment plays a major role in determining tumor survival and growth. In particular, the role of immunity in eliminating or promoting tumor growth has been now clearly recognized (3–5).

The complex nature of tumorigenesis requires the identification of different therapeutic targets and the implementation of different therapeutic approaches, including drugs/strategies that could overcome resistance after treatment. The immunotherapeutic approaches with immune checkpoint inhibitors are an example of treatment that does not induce resistance, as its target is not the tumor but the re-activation of specific tumoricidal immune cells (6, 7). Thus, the development of anti-PD-1 and anti-PD-L1 immune checkpoint inhibitors has opened the way to novel anti-tumor approaches that go beyond inhibiting oncogenic signals and address the tumor microenvironment as a whole. In this context, AXL is an attractive target for designing future treatment targets.

AXL is a receptor tyrosine kinase of the TAM (TYRO3-AXL-MER) family (8–10). High AXL expression in tumor cells promotes aggressiveness, metastatic capacity and refractoriness to drug treatment (11, 12). In parallel, it was observed that the expression of AXL and other TAM receptors is higher in macrophages polarized towards the M2 anti-inflammatory pro-tumoral functional phenotype and that activation of AXL can induce M2 macrophages polarization (13–16). Since the M2 phenotype is typical of pro-tumoral tumor-associated macrophages, this points to AXL inhibition as a double target to reduce the aggressiveness of tumor cells and the tumor-promoting activity of the innate immune cells (macrophages). Importantly, AXL has been identified as a receptor used by several viruses (Zika, Dengue, RSV, JEV, SARS-CoV-2) for entry into host cells and spreading infections (17–20). Thus, inhibition of AXL in infectious diseases could have the double advantage of blocking virus entry and allowing protective macrophage activation (M1 polarization), by inhibiting M2 development. For these reasons, new drugs targeting AXL are being developed. An overview of AXL role in tumor and immune cells and the potential benefits of its inhibition are presented in **Figure 1**.

**Figure 1:**
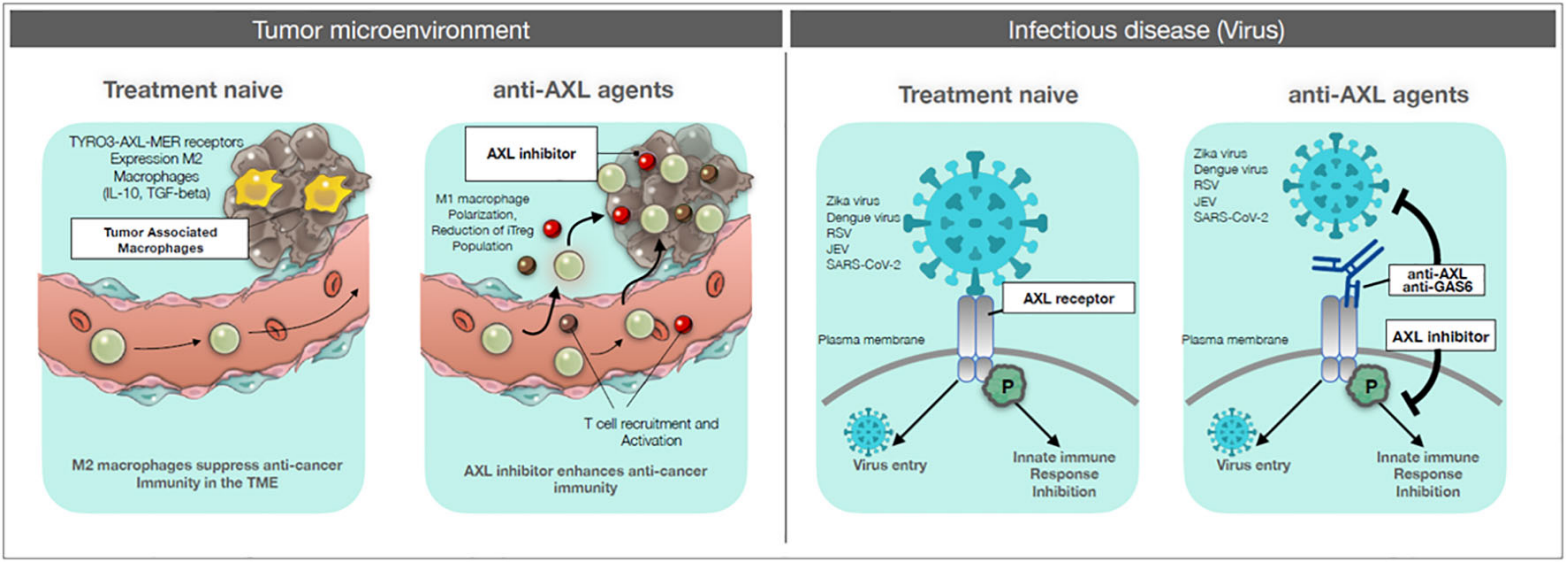
AXL targeting drugs on cancer and infectious diseases. Left panels: Tumor microenvironment. Left: treatment-naïve: circulating monocytes and tissue-resident macrophages are recruited to the tumor site. These tumor-associated macrophages are polarized towards an M2 functional phenotype, which can suppress anti-tumor response and promote tumor growth. Right: anti-AXL agents: by blocking the signaling pathway of AXL with anti-AXL agents, recruited monocytes and macrophages can be polarized towards the inflammatory, immunostimulatory and cytocidal M1 phenotype, thereby allowing the development of anti-tumor immune responses and also acting as anti-tumor killer cells. Right panels: Infectious diseases. Left: treatment-naïve: viruses (Zika, Dengue, RSV, JEV, SARS-CoV-2) can gain entry into the host cell through interaction with an AXL receptor, thereby efficiently infecting the host. In addition, viral binding can induce an AXL-mediated inhibitory signaling for innate immune responses. Right: anti-AXL agents: anti-AXL agents that bind to the extracellular domain of AXL can block virus binding and subsequent infection. Also, anti-AXL agents that block the virus-induced AXL inhibitory signaling can restore efficient innate immunity against the viral infection.

New technologies to suppress AXL functions include inhibition of downstream signaling with antibodies or blockade of GAS6, the ligand of AXL, and the use of small molecules that can inhibit AXL phosphorylation. The state-of-the-art of the use of AXL inhibitors in cancer therapy is reported by Tanaka et al., who have extensively described the AXL signaling pathway and listed the most promising AXL inhibitors that have been developed clinically or preclinically (21). The review presents the anti-tumor mechanisms of AXL inhibitors, and the i privement of therapeutic efficacy and immunoactivity of chemotherapy currently use in clinical practice (21).

The contribution of AXL to immune evasion underlines the importance of its inhibition in anti-tumor therapies. GAS6/AXL signaling can participate to evasion from anti-tumor immune response within the tumor microenvironment in multiple ways (Son and Jeong). These include modulation of MHC-1 and PD-L1 expression, production of immunosuppressive cytokines, increased recruitment of myeloid derived suppressor cells and decreased infiltration of T cells, inhibition of apoptosis, promotion of CTLA4 and Foxp3 expression in Treg cells and of their immunosuppressive activity, and regulation of tumor associated innate immune cells. The use of AXL inhibitors, both in preclinical studies and in clinical trials, provides valuable information on the modulation of GAS6/AXL-mediated immune evasion (Son and Jeong). Not only AXL but also other receptors of the family, such as TYRO3, are strongly involve in the regulation of tumor cell apoptosis, as demonstrated in colon carcinoma *in vitro* and *in vivo* models by using a specific inhibitor (Kim et al.).

The potential application of AXL inhibitor as a single agent or in combination with immunotherapy in patients with lung cancer has been investigated based on clinical treatment results and treatment advantages (Sang et al.). AXL inhibitors have shown therapeutic benefits in combination with chemotherapy to increase survival rate through enhanced activation of immune cells (Lee et al.). However, detailed anti-tumor effects of AXL signaling pathway is yet to be understood.

While the current development of AXL inhibitors offers promising opportunities for effective therapeutic intervention against cancer, future research should aim at fully clarifying the effects of AXL and, more in general, TAM inhibitors. This should start from a complete understanding of the physiological role of these receptors and the assessment of their pathology-linked molecular and functional variations. On these grounds, AXL inhibition can be precisely targeted, to attain high therapeutic efficacy and limited side effects. The possibility of modulating AXL functions in macrophages and in virus-host interactions opens a large spectrum of therapeutic/preventive possibilities beyond cancer.

## Author contributions

KP wrote the editorial, RM and DB contributed to writing the manuscript and critically revised it. All authors approved the submitted version.

## References

[1] Falzone L Salomone S Libra M. Evolution of cancer pharmacological treatments at the turn of the third millennium. Front Pharmacol (2018) 9:1300. 30483135 10.3389/fphar.2018.01300 PMC6243123

[2] Huang L Jiang S Shi Y. Tyrosine kinase inhibitors for solid tumors in the past 20 years (2001-2020). J Hematol Oncol (2020) 13:143. 33109256 10.1186/s13045-020-00977-0 PMC7590700

[3] Galli F Aguilera JV Palermo B Markovic SN Nistico P Signore A. Relevance of immune cell and tumor microenvironment imaging in the new era of immunotherapy. J Exp Clin Cancer Res (2020) 39:89. 32423420 10.1186/s13046-020-01586-y PMC7236372

[4] Lei X Lei Y Li JK Du WX Li RG Yang J. Immune cells within the tumor microenvironment: Biological functions and roles in cancer immunotherapy. Cancer Lett (2020) 470:126–33. 10.1016/j.canlet.2019.11.009 31730903

[5] Mao X Xu J Wang W Liang C Hua J Liu J. Crosstalk between cancer-associated fibroblasts and immune cells in the tumor microenvironment: new findings and future perspectives. Mol Cancer (2021) 20:131. 34635121 10.1186/s12943-021-01428-1 PMC8504100

[6] Zhang Y Zhang Z. The history and advances in cancer immunotherapy: Understanding the characteristics of tumor-infiltrating immune cells and their therapeutic implications. Cell Mol Immunol (2020) 17:807–21. 10.1038/s41423-020-0488-6 PMC7395159 32612154

[7] Jacob JB Jacob MK Parajuli P. Review of immune checkpoint inhibitors in immuno-oncology. Adv Pharmacol (2021) 91:111–39. 10.1016/bs.apha.2021.01.002 34099106

[8] Wu G Ma Z Cheng Y Hu W Deng C Jiang S. Targeting Gas6/TAM in cancer cells and tumor microenvironment. Mol Cancer (2018) 17:20. 29386018 10.1186/s12943-018-0769-1 PMC5793417

[9] Burstyn-Cohen T Maimon A. TAM receptors, phosphatidylserine, inflammation, and cancer. Cell Commun Signal (2019) 17:156. 31775787 10.1186/s12964-019-0461-0 PMC6881992

[10] Ghosh Roy S. TAM receptors: a phosphatidylserine receptor family and its implications in viral infections. Int Rev Cell Mol Biol (2020) 357:81–122. 33234246 10.1016/bs.ircmb.2020.09.003

[11] Shieh YS Lai CY Kao YR Shiah SG Chu YW Lee HS. Expression of axl in lung adenocarcinoma and correlation with tumor progression. Neoplasia (2005) 7:1058–64. 10.1593/neo.05640 PMC1501169 16354588

[12] Chai ZT Zhang XP Ao JY Zhu XD Wu MC Lau WY. AXL overexpression in tumor-derived endothelial cells promotes vessel metastasis in patients with hepatocellular carcinoma. Front Oncol (2021) 11:650963. 34123800 10.3389/fonc.2021.650963 PMC8191462

[13] Fujimori T Grabiec AM Kaur M Bell TJ Fujino N Cook PC. The axl receptor tyrosine kinase is a discriminator of macrophage function in the inflamed lung. Mucosal Immunol (2015) 8:1021–30. 10.1038/mi.2014.129 PMC4430298 25603826

[14] Chiu KC Lee CH Liu SY Chou YT Huang RY Huang SM. Polarization of tumor-associated macrophages and Gas6/Axl signaling in oral squamous cell carcinoma. Oral Oncol (2015) 51:683–9. 10.1016/j.oraloncology.2015.04.004 25910588

[15] Chen SY Chiang CF Chiu KC Cheng CW Huang SM Chen PH. Macrophage phenotypes and Gas6/Axl signaling in apical lesions. J Dent Sci (2019) 14:281–7. 10.1016/j.jds.2018.12.002 PMC6739459 31528256

[16] Jimenez-Garcia L Mayer C Burrola PG Huang Y Shokhirev MN Lemke G. The TAM receptor tyrosine kinases axl and mer drive the maintenance of highly phagocytic macrophages. Front Immunol (2022) 13:960401. 35967387 10.3389/fimmu.2022.960401 PMC9373726

[17] Shibata T Makino A Ogata R Nakamura S Ito T Nagata K. Respiratory syncytial virus infection exacerbates pneumococcal pneumonia *via* Gas6/Axl-mediated macrophage polarization. J Clin Invest (2020) 130:3021–37. 10.1172/JCI125505 PMC7260035 32364537

[18] Wang S Qiu Z Hou Y Deng X Xu W Zheng T. AXL is a candidate receptor for SARS-CoV-2 that promotes infection of pulmonary and bronchial epithelial cells. Cell Res (2021) 31:126–40. 10.1038/s41422-020-00460-y PMC7791157 33420426

[19] Wang ZY Zhen ZD Fan DY Qin CF Han DS Zhou HN. Axl deficiency promotes the neuroinvasion of Japanese encephalitis virus by enhancing IL-1α production from pyroptotic macrophages. J Virol (2020) 94:e00606–20. 10.1128/JVI.00602-20 PMC7431807 32611752

[20] Xie S Zhang H Liang Z Yang X Cao R. AXL, an important host factor for DENV and ZIKV replication. Front Cell Infect Microbiol (2021) 11:575346. 33954117 10.3389/fcimb.2021.575346 PMC8092360

[21] Tanaka M Siemann DW. Therapeutic targeting of the Gas6/Axl signaling pathway in cancer. Int J Mol Sci (2021) 22:9953. 34576116 10.3390/ijms22189953 PMC8469858

